# Detection of the sentinel lymph node with hybrid tracer (ICG-[^99m^Tc]Tc-albumin nanocolloid) in intermediate- and high-risk endometrial cancer: a feasibility study

**DOI:** 10.1186/s13550-021-00863-x

**Published:** 2021-12-14

**Authors:** Nuria Sánchez-Izquierdo, Sergi Vidal-Sicart, Francisco Campos, Aureli Torné, Martina Aida Angeles, Federico Migliorelli, Meritxell Munmany, Adela Saco, Berta Diaz-Feijoo, Ariel Glickman, Jaume Ordi, Andrés Perissinotti, Marta del Pino, Pilar Paredes

**Affiliations:** 1 Department of Nuclear Medicine, Hospital Clínic of Barcelona, Villarroel 170, 08036 Barcelona, Spain; 2 Institute Clinic of Gynecology, Obstetrics, and Neonatology, Hospital Clínic of Barcelona, Barcelona, Spain; 3 Department of Pathology, Hospital Clínic of Barcelona, Barcelona, Spain; 4 Department of Surgical Oncology, Institut Claudius Regaud, Institut Universitaire du Cancer de Toulouse - Oncopole, Toulouse, France; 5 Department of Gynecology and Obstetrics, Centre Hospitalier Intercommunal des Vallées de l’Ariège, Saint-Jean-de-Verges, France; 6 Institut d’Investigacions Biomèdiques August Pi I Sunyer (IDIBAPS), Barcelona, Spain; 7 Faculty of Medicine, University of Barcelona, Barcelona, Spain; 8 ISGlobal, Barcelona Institute for Global Health, Hospital Clínic-Universitat de Barcelona, 08036 Barcelona, Spain

**Keywords:** Hybrid tracer (ICG-[^99m^Tc]Tc-albumin nanocolloid), Sentinel lymph node, Endometrial cancer, Lymphatic mapping, Radiotracer, ICG

## Abstract

**Purpose:**

Indocyanine green (ICG) is frequently used for the detection of the sentinel lymph node (SLN) in gynecology, but it carries the loss of the presurgical SLN mapping provided by [^99m^Tc]-based colloids. Hybrid tracers such as ICG-[^99m^Tc]Tc-albumin nanocolloid combine the benefits of both components. The aim of this study was to evaluate the feasibility and applicability of this hybrid tracer injected by transvaginal ultrasound-guided myometrial injection of radiotracer (TUMIR) approach in the detection of SLNs in patients with intermediate- and high-risk EC.

**Methods:**

Fifty-two patients with intermediate- and high-risk EC underwent SLN biopsy after injection of a hybrid tracer using the TUMIR approach, followed by pelvic and paraaortic lymphadenectomy. SLNs were detected preoperatively by lymphoscintigraphic study and intraoperatively by gamma probe and near-infrared (NIR) optical laparoscopic camera.

**Results:**

Preoperative lymphatic drainage was obtained in 69% and intraoperative detection in 71.4% of patients. A total of 146 SLNs (4.17 SLNs/patient) were biopsied. Pelvic bilateral detection was observed in 57% of the women and paraaortic drainage in 34% of the patients. The radioactive component allowed the detection of SLN in 97.1% of the patients, while the fluorescent component detected 80%. In more than 17% of the patients with intraoperative detection, SLNs were detected only by the radioactive signal. Lymph node metastasis was identified in 14.3% of patients submitted to SLNB. The sensitivity and negative predictive value for metastatic involvement were 100%.

**Conclusion:**

TUMIR injection of a hybrid tracer in patients with intermediate- and high-risk EC combines the benefits of the radiotracer and the fluorescence methods with a single tracer. The method increases the paraaortic detection rate and allows a potential increase in SLN detection. Notwithstanding, based on our findings, the radioactive component of the hybrid tracer cannot be obviated.

## Introduction

Endometrial cancer (EC) is the most frequent gynecological malignancy in high-income countries. ECs are classified as low, intermediate and high risk based on their risk of lymph node and distant metastases. As in most solid tumors, lymph node infiltration is the most important prognostic factor, and therefore, systematic pelvic and paraaortic lymphadenectomy is indicated in high-risk tumors and is considered in those of intermediate risk [1]. However, the role of systematic lymphadenectomy has recently been questioned because it has a significant associated morbidity and results in an overtreatment of up to 80% of women [2]. Sentinel lymph node biopsy (SLNB) has been developed as an alternative to systematic lymphadenectomy, associated with a low morbidity rate. Furthermore, SLN ultrastaging, which involves serial sections and the use of immunohistochemical stains, allows increasing the sensitivity for metastasis. The guidelines of the National Comprehensive Cancer Network recommend SNLB in patients with low-risk EC [3]. Recent evidence indicates that SLNB achieves a detection rate of 89% and a false negative rate of 11.5% in patients with intermediate- and high-risk EC [4]. However, its indication in these women with intermediate- and high-risk EC is still under debate.

Traditionally, SLNB in patients with gynecological tumors has been performed using a technetium-based radiotracer ([^99m^Tc]Tc-albumin nanocolloid), either alone or combined with blue dye. In these cases, the radiotracer allows obtaining preoperative lymph node mapping, which increases the detection of SLN [5]. Nonetheless, the SLN detection rate is lower in patients with EC than in other gynecological tumors. Reduced SLN detection in these patients has been associated with older age, tumor size bigger than 2 cm and low radiotracer volumes [6]. The combination with blue dye does not seem to significantly increase the rate of detection in these patients with EC [7, 8].

To solve these problems, the use of fluorescent, non-radioactive tracers has been introduced. Among these tracers, indocyanine green (ICG), which can be detected intraoperatively, has been used as a single tracer in gynecological tumors of the endometrium [9, 10], cervix [11, 12] and vulva [13]. However, unlike radiotracer, ICG is not retained by the macrophages of the lymph nodes, and therefore, the injection must be performed intraoperatively due to its rapid drainage, precluding obtaining a preoperative lymph node map for planning surgery.

In the last decade, hybrid tracers (ICG-[^99m^Tc]Tc-albumin nanocolloid) have been developed. These hybrid tracers combine the advantages of the two modalities [14–16]: the radiotracer allows presurgical lymphatic mapping and provides an acoustic signal through the gamma detector probe, whereas ICG allows the visualization of the SLN during surgery. This hybrid tracer has shown to be more efficient and accurate to localize the SLN compared with blue dye or the two components (ICG and radiotracer) used alone [14–16] in urological neoplasms such as cancers of the prostate gland [17] and penis [14]. The hybrid tracer has also been successfully used in gynecological neoplasms, such as vulvar [18] and cervical cancer [19], with a high rate of SLN detection (96–100%), but has not been evaluated in EC.

The aim of this study was to evaluate the feasibility of using a single hybrid tracer and its performance to detect SLN in patients with high and intermediate-risk EC.

## Material and methods

### Patients and study design

Patients with preoperative diagnosis of intermediate and high-risk EC submitted to SLNB and clinically indication in our center of complete pelvic and paraaortic lymphadenectomy between 2014 and 2019 were included in this study. The patients fulfilled at least one of the following inclusion criteria: (1) unfavorable histology (serous, clear cell or grade 3 endometrioid adenocarcinoma); (2) myometrial invasion ≥ 50% suspected by imaging techniques (magnetic resonance imaging (MRI) or 3D ultrasound); (3) involvement of the cervical stroma confirmed by biopsy or suspected by imaging techniques.

Patients with the following criteria were excluded from our study: (1) contraindication for surgical staging; (2) metastatic disease suspected in the preoperative evaluation by computed tomography (CT) or MRI or confirmed histologically; (3) previous surgery or radiotherapy in the pelvic or paraaortic regions.

This retrospective study from prospectively collected data was approved by our institutional review board (HCB/2019/0574), and written informed consent was obtained from all the patients included in the study prior to the administration of the hybrid tracer.

### Injection of the hybrid tracer

The hybrid tracer was prepared in the radiopharmacy unit according to the usual methodology of our center [19]. Between 18 and 24 h before surgery, the patients were injected with a dose of 222 MBq (6 mCi) at a total volume of 4 ml of hybrid tracer. Two ml of the hybrid tracer plus 1 ml of physiological saline was injected into the anterior and posterior part of the myometrium to increase interstitial pressure in the tissue, separated by 1 ml of air between the two injections to avoid in vivo dilution of the hybrid tracer.

**Fig. 1:**
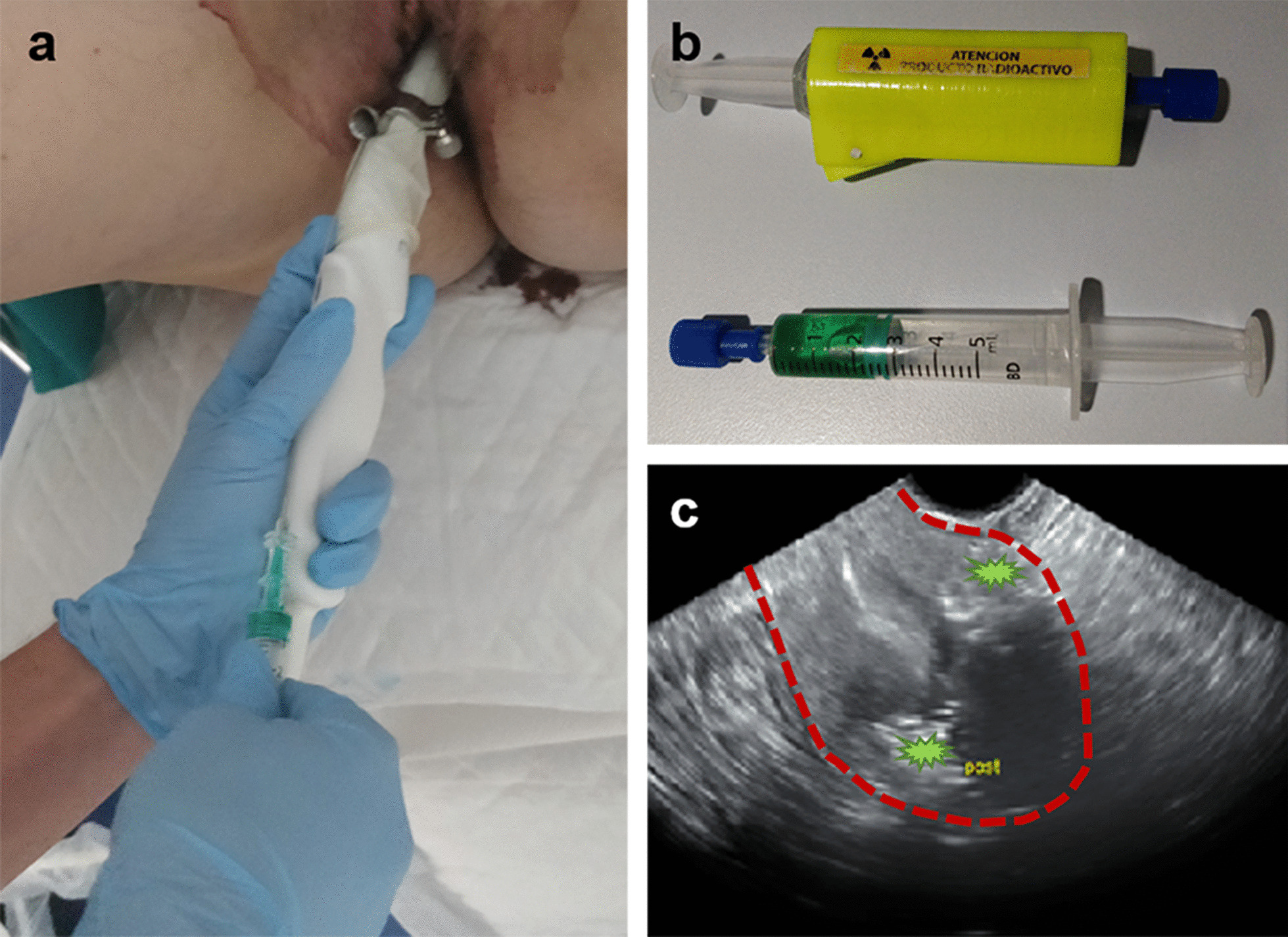
Transvaginal ultrasound myometrial injection of the hybrid tracer (**a**). Hybrid tracer (ICG-[^99m^Tc]Tc-nanocolloid) prepared for the injection (**b**). Tracer accumulation (green asterisk) in the anterior and posterior walls of the myometrium. Intermittent red line delimitates the uterus. Red double-headed arrow indicates the endometrium (**c**)

This injection technique used was the transvaginal ultrasound-guided myometrial injection of radiotracer (TUMIR) approach, as previously described [6, 20]. An Aspen (Siemens-Acuson Inc., MountainView, CA, USA) or Voluson ultrasound image equipment (Voluson v730Expert, General Electric, Germany), equipped with a vaginal probe coupled with a caliber 20 biopsy needle guide (Gallini Medical Devices, Mantova, Italy), was used for injection of the hybrid tracer. All the procedures were performed by an expert gynecologist together with a specialist in nuclear medicine (Fig. 1).

### Preoperative lymphoscintigraphy

Following the injection of the hybrid tracer, planar abdominal-pelvic images (matrix of 256 × 256, anterior and lateral views of 300 s/frame) were obtained using a single-head gamma camera (E-Cam, Siemens, Erlangen, Germany) or dual-head gamma camera (Infinia™ Hawkeye™ 4; GE Healthcare Milwaukee, WI, USA), equipped with a low-energy high-resolution collimator. The images were obtained at 30 min and at 2–4 h after the injection of the hybrid tracer.

Afterward, single photon emission computed tomography (SPECT) images with CT for correction of attenuation and posterior fusion of the reconstructed image were acquired with two different dual-head gamma camera (Infinia™ Hawkeye™ 4; GE Healthcare Milwaukee, WI, USA) (120 projections in a non-circular 360° orbit, 15 s/projection in the matrix of 128 × 128 with a pixel size of 3.16 × 3.16 mm^2^) using a CT image (matrix size 512 × 512, 140 kV and 2.5 mAs) and a Symbia Intevo Bold (Siemens Healthineers, Erlangen, Germany) gamma camera was used (120 projections in a non-circular 360° orbit, 30 s/projection, in a matrix of 128 × 128 with a pixel size of 3.30 × 3.30 mm^2^), with CT image (matrix size 512 × 512, 130 kV and 4D CARE Dose).

**Fig. 2:**
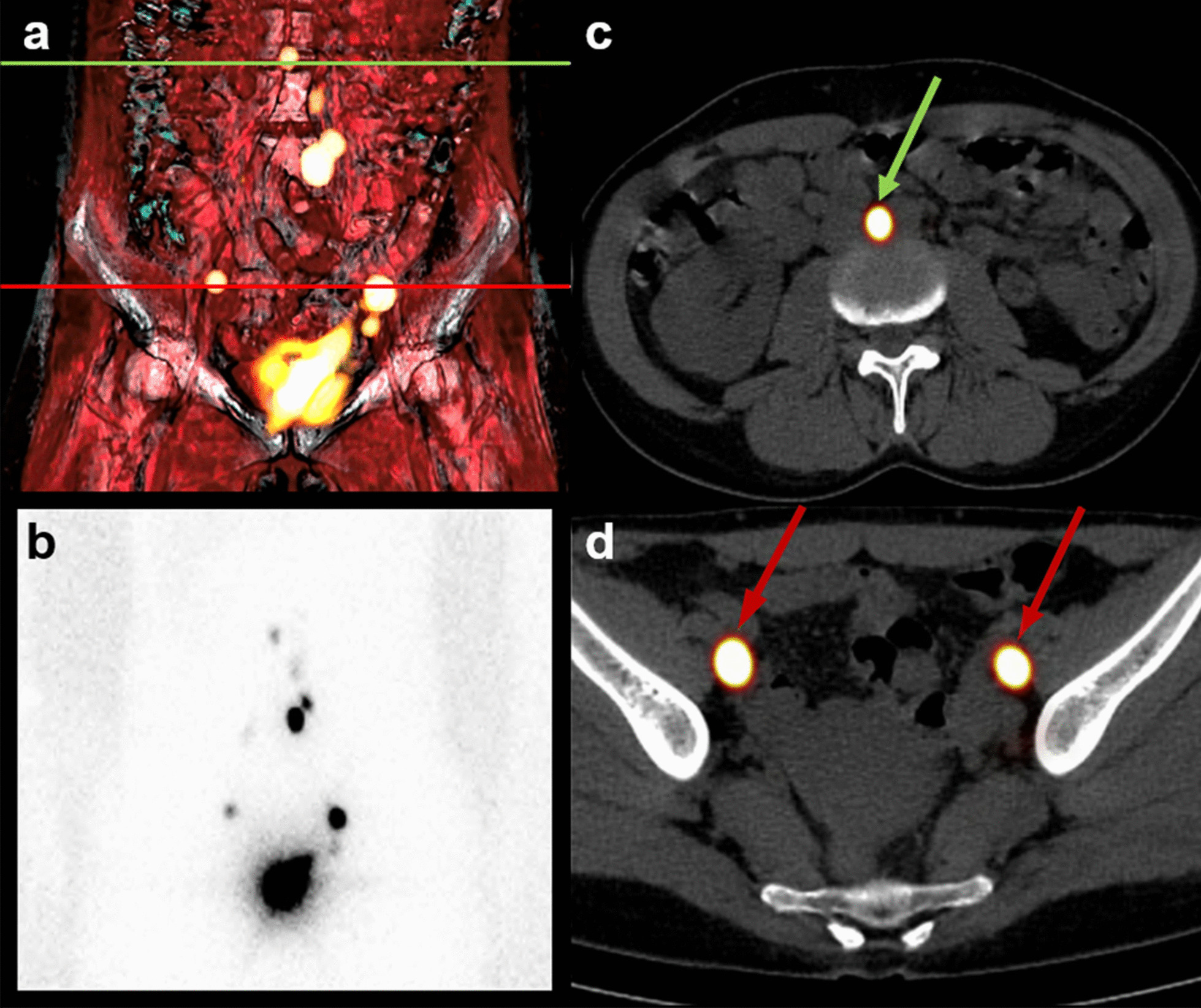
Planar and SPECT/CT lymphoscintigraphy performed 2 h after TUMIR injection of hybrid tracer (ICG-[^99m^Tc]Tc-nanocolloid). Volumetric reconstruction of SPECT/CT (**a**) and planar lymphoscintigraphy (**b**) reveal drainage to the bilateral pelvic and paraaortic regions. Axial fused SPECT/CT images showing radioactive SLNs corresponding to a paraaortic node (green arrow) (**c**) and bilateral external iliac SLNs (red arrows) (**d**). SPECT/CT: single photon emission computed tomography; TUMIR: transvaginal ultrasound-guided myometrial injection of radiotracer; SLNs: sentinel lymph node

In addition, volumetric reconstructions were performed using the Osirix Dicom viewer (Pixmeo SARL, Geneva, Switzerland) in an operating system based on Unix (MAC OS X, MacPro; Apple, Cupertino, CA) for obtaining a tridimensional presentation and improving anatomical localization of the SLNs (Fig. 2).

The images were examined by two specialists in Nuclear Medicine and were discussed with the surgical team prior to surgery.

### Intraoperative SLN detection

In all the patients, the detection of SLNs was made by laparoscopic surgery. The laparoscopic surgery was started by an intraperitoneal approach to rule out carcinomatosis. Then, a left retroperitoneal access was created to first localize the paraaortic SLNs.

For intraoperative localization of the SLN, a laparoscopic gamma ray detector probe was used (Navigator; USSC, Norwalk, CT, USA), which is compatible with a laparoscopic trocar of 12 mm in diameter. For the detection of fluorescence, a specialized laparoscopic optical camera was used, which included a near-infrared (NIR) image option (Karl Storz Image1 S™; Karl Storz, Tuttlingen, Germany). This system allows obtaining images with white light and fluorescence in the NIR spectrum according to in vivo needs.

**Fig. 3:**
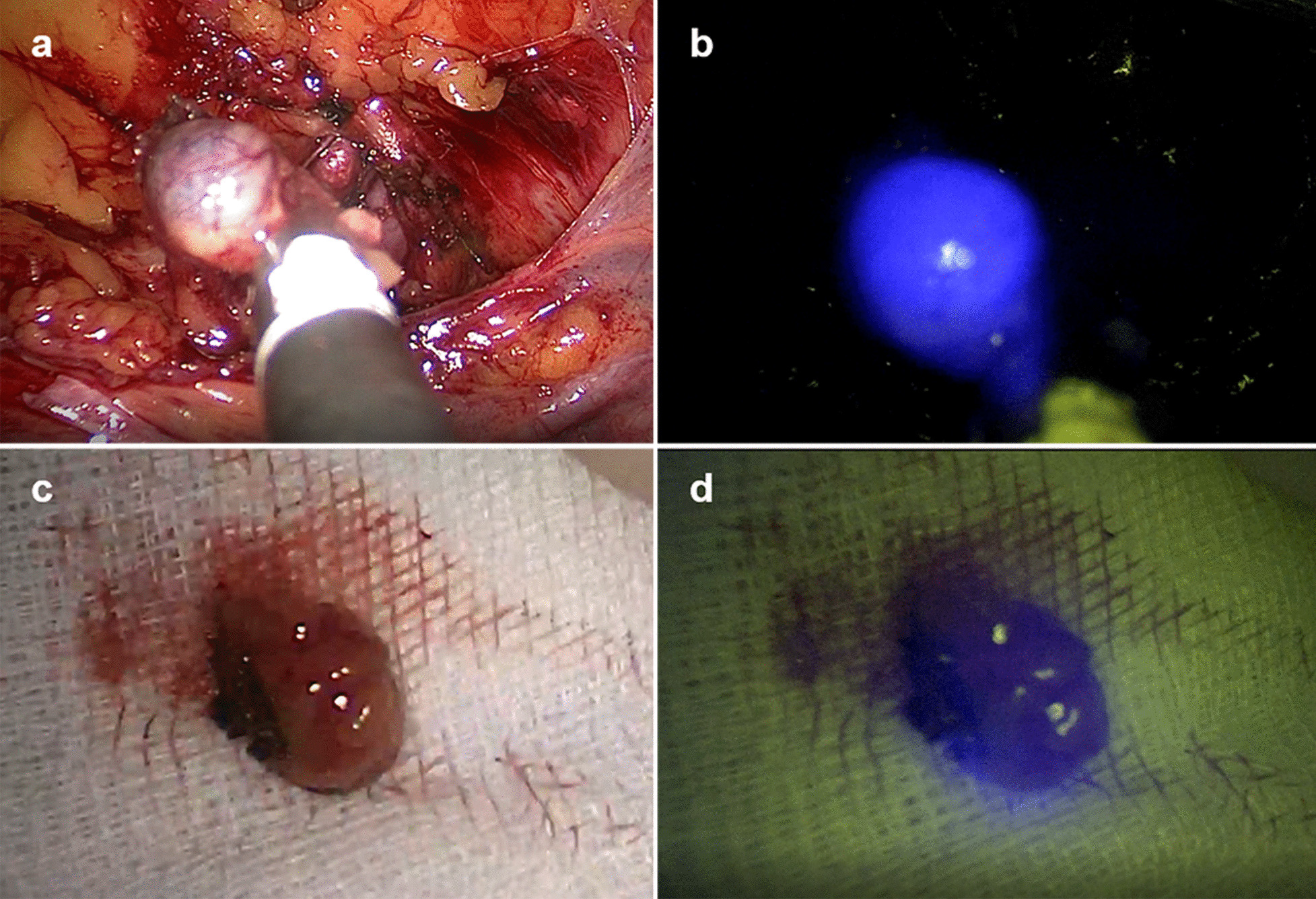
Intraoperative detection of SLNs by gamma probe and NIR optical camera: Combined laparoscopic sentinel node visualization with white light image (**a**) and multispectral fluorescence imaging (shown in blue) (**b**). Ex vivo images of the SLN biopsied with white light (**c**) and multispectral fluorescence (shown in blue) (**d**). SLNs: sentinel lymph node. NIR: near infrared

After SLNB, the number of counts emitted by the radiotracer and the intensity of the luminous emission of NIR ex vivo were collected using the same devices described for the in vivo detection (Fig. 3).

A lymph node was considered a SLN when: (a) it was the first node visualized in the lymphoscintigraphy or showed greater activity in the late planar images or was visualized in the SPECT/CT in a region other than in the planar lymphoscintigraphy; (b) it was the most active node during surgery according to the gamma probe or previous lymphoscintigraphy; or (c) it was fluorescent. Each side of the pelvis and the paraaortic area were considered independent lymphatic territories according to the Memorial Sloan Kettering Cancer Center algorithm [21]. The exact location of each SLN in relation to the pelvic, vena cava or aorta vessels was recorded. The number of SLN excised, their localization and their intraoperative detection signal were reported.

### Paraaortic lymphadenectomy, pelvic SLNB and hysterectomy

After selective excision of paraaortic SLNs, systematic paraaortic lymphadenectomy was carried out. The procedure included removal of the lymph nodes located at the presacral, aortic bifurcation, precaval, preaortic and paraaortic areas, both below and above the inferior mesenteric artery up to the level of the left renal vein as the upper limit of dissection.

Lymphadenectomy continued through a transperitoneal approach to perform a selective excision of pelvic SLNs. The pelvic regions were carefully scanned with the gamma probe, angled laterally to avoid detection of radioactivity at the injection site. Then, a bilateral transperitoneal pelvic lymphadenectomy was performed, including the removal of external and internal iliac, obturator fossa, and common iliac lymph nodes. Finally, a total vaginal hysterectomy and bilateral salpingo-oophorectomy assisted by laparoscopy were performed.

### Histological evaluation of the lymph nodes

The SLNs were cut into 2-mm-thick serial sections, submitted completely for histology and routinely embedded in paraffin. A first four-micrometer-thick section was stained with hematoxylin and eosin (H&E) and examined under a light microscope. If this first section was negative, four additional pairs of sections were performed at an interval of 200 μm (ultrastaging). Of each pair, one section was stained with H&E and the other using immunohistochemistry for cytokeratin 7 (Dako Pathology, Agilent, Santa Clara, CA, USA). Immunohistochemical studies were performed with the automated immunohistochemical system Autostainer Link 48®, using the EnVision system (Dako).

The lymphadenectomy specimens were fixed in neutral-buffered formalin and macroscopically dissected to isolate all lymph nodes, which were cut into 2-mm-thick sections following their largest diameter and routinely processed. Four-micrometer-thick histological sections were obtained with a microtome, which were stained with H&E and examined under a light microscope.

Metastatic involvement was defined as at least one lymph node (either SLN or one non-SLN) positive for metastases detected either in the evaluation of the H&E and/or the immunohistochemical section (in case of SLN). When present, the size of the metastasis was recorded. Isolated tumor cells and metastatic involvement less than 2 mm (micrometastases) were considered as low-volume metastases.

### Statistical analysis

Categorical variables are described as absolute numbers and percentage, while continuous features are shown as mean and standard deviation (SD).

The number of SLNs retrieved during surgery was compared with the number of SLN identified in the planar lymphoscintigraphy and SPECT/CT. We also compared the number of SLN detected by each of the tracers, individually and simultaneously (ICG/Radiotracer/ICG + Radiotracer).

Categorical variables were evaluated by the Fisher exact test and continuous variables by the Student’s t-test. The paired Student’s t-test was used to evaluate the preoperative and postoperative detection rates.

The accuracy analysis of the hybrid detection of the SLN was carried out including exclusively the women with successful SLN resection (meaning that at least one SLN was intraoperatively identified and excised). The histological result of the SLN was contrasted with the result of the histopathological analysis of the nodes retrieved during lymphadenectomy. With these data, sensitivity, specificity and the positive predictive value (PPV) and negative predictive value (NPV) of the histological results of the SLN were calculated, as well as their respective 95% confidence intervals (CI), using the Wilson’s method. Differences were considered significant at a 5% bilateral level, and all analyses were performed using Stata 13.1 (StataCorp, Texas).

## Results

During the study period, 83 patients were cared for in our center with inclusion criteria for our study. In 22 cases, there was no ICG available to label the hybrid radiotracer, and 9 patients refused the hybrid tracer to choose the conventional radiotracer. A total of 52 patients were included in the analysis with preoperative intermediate (33 women) or high-risk (19 women) EC, assessed according to ESMO-ESGO-ESTRO Consensus Conference on EC Classification. The mean age of the patients was 63.6 years (SD 10.3). The histological characteristics and International Federation of Gynecology and Obstetrics stage of the patients included in the study are summarized in Table 1. There were no adverse reactions either during or after injection of the hybrid tracer.

**Table 1:** Postoperative histological characteristics of the 52 patients included in the study

**Histological type and grade**	
Endometrioid	38 (73.0%)
Grade 1	9 (23.7%)
Grade 2	20 (52.6%)
Grade 3	9 (23.7%)
Serous	7 (13.5%)
Clear cell	5 (9.6%)
Mixed	2 (3.8%)
**Histological tumor size**	
< 4 cm	27 (51.9%)
≥ 4 cm	25 (47.2%)
Postoperative FIGO* stage	
IA	25 (47.2%)
IB	12 (23.0%)
II	4 (7.5%)
IIIA	0 (0.0%)
IIIB	0 (0.0%)
IIIC1	5 (9.4%)
IIIC2	3 (5.7%)
IVA	0 (0.0%)
IVB	3 (5.7%)

^*^FIGO: International Federation of Gynecology and Obstetrics (2009)

### Preoperative detection: lymphoscintigraphy (planar and SPECT/CT images)

Drainage toward the pelvic and/or paraaortic lymph node chains was observed in 48.1% (25/52) of the patients in the planar lymphoscintigraphy images and in 69.2% (36/52) in the tomographic images of SPECT/CT. Among the women in whom SLN was detected, bilateral pelvic SLN was visualized in 55.6% (20/36). In none of the women, the drainage was exclusively paraaortic. Table 2 shows the distribution of the drainage.

**Table 2:** Comparison of preoperative detection rate for different techniques and distribution of pelvic and paraaortic SLNs identified in planar lymphoscintigraphic and SPECT/CT images and intraoperative detection

	Planar lymphoscintigraphic DR (n, %)	SPECT/CT DR (n, %)	Intraoperative detection rate (n, %)
	25/52 (48.1%)	36/52 (69.2%)	35/49 * (71.4%)
**Area of SLN detection**			
Pelvis (exclusive)	13 (52.0%)	23 (63.9%)	23 (65.7%)
Paraaortic (exclusive)	0 (0.0%)	0 (0.0%)	0 (0.0%)
Pelvis and paraaortic area	12 (48.0%)	13 (36.1%)	12 (34.3%)
**Side of pelvis**			
Left	2 (8.0%)	3 (8.3%)	3 (8.6%)
Right	7 (28.0%)	13 (36.1%)	12 (34.3%)
Bilateral	16 (64.0%)	20 (55.6%)	20 (57.1%)

DR: preoperative detection rate (drainage on lymphoscintigraphy); SLNs: sentinel lymph node. SPECT/CT: single photon emission computed tomography

^*^During surgery, three of the 52 patients showed peritoneal carcinomatosis, so SLN biopsy was ruled out. That results in 49 patients in whom SLN biopsy was attempted

In 30.8% (16/52) of the patients, drainage was not observed in the presurgical images. In three of these 16 women (6%), peritoneal diffusion was observed; in other three (6%), there was an important uptake in the bone marrow, and the remaining 10 patients (19%) showed an absence of lymphatic drainage of the hybrid tracer.

Among the 36 patients showing drainage, 79 SLNs were observed in the planar images, with a mean of 2.19 (SD 1.92) per patient, and 138 SLNs were visualized in the SPECT/CT images with a mean of 3.83 (SD 2.75) per patient (*p* < 0.05).

### Intraoperative detection: surgery

**Fig. 4:**
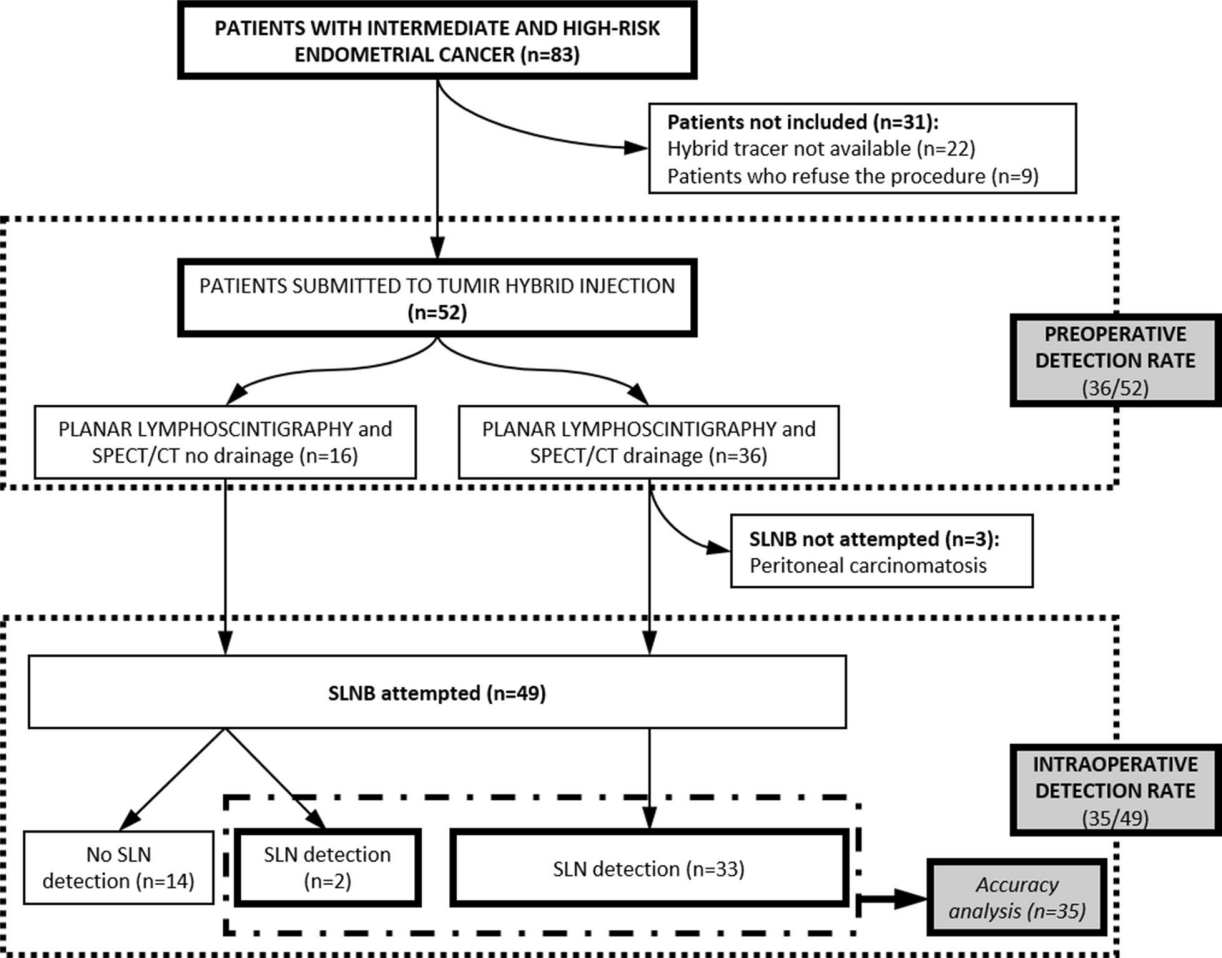
Flow chart of patients available for analysis and included in the study. SPECT/CT: single photon emission computed tomography. SLNB: sentinel lymph node biopsy; SLN: sentinel lymph node

During surgery, three patients (belonging to the group of patients with drainage) showed peritoneal carcinomatosis, so SLNB was ruled out. That results in 49 patients in whom SLNB was attempted. Among the 16 women without preoperative drainage and who underwent surgery, SLNs were intraoperatively detected in two patients. In the remaining 14 patients, the absence of lymphatic drainage was confirmed (26.9%). At least one SLN was intraoperatively detected in 35 patients of the 49, which means an intraoperative detection rate of 71.4% (35/49). These 35 women will comprise the group of patients for the accuracy analysis. These findings are shown in the flow chart of Fig. 4.

**Fig. 5:**
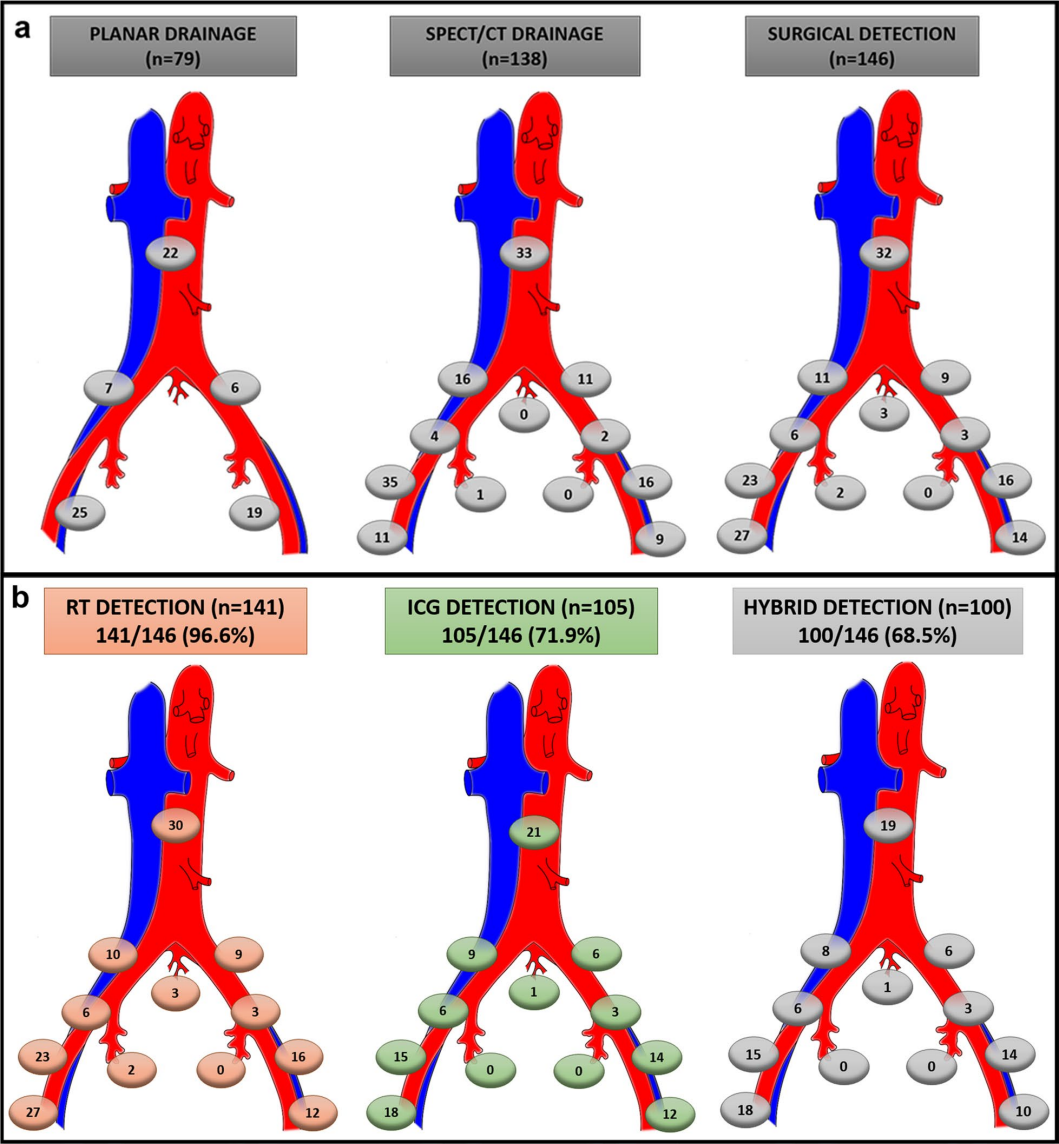
Topographic distribution of SLNs identified in different anatomical areas on preoperative examination and surgery (a) and depending on the tracer detecting by gamma- and/or NIR-emission (b): radiotracer, indocyanine green and hybrid detection (radiotracer and indocyanine green). SLNs: sentinel lymph node. NIR: near infrared; SPECT/CT: single photon emission computed tomography; RT: radiotracer; ICG: indocyanine green

A total of 146 SLNs were excised with a mean of 4.17 (SD 2.81) SLN per patient (146/35), with bilaterality of 57.1% (20/35). Paraaortic SLNs were biopsied in 34.3% (12/35) of the patients. No isolated paraaortic SLNs were biopsied. Figure 5a shows the topographic distribution of the SLNs.

In the *analysis of SLN detection by tracer*, of the 146 SLNs excised, 96.6% (141/146) showed radioactivity signal, and 71.9% (105/146) showed fluorescence signal. Among the 146 SLNs, 68.5% (100/146) of the SLNs biopsied were detected by both components. In 28.1% (41/146), the SLNs were detected by the radioactive signal, but we were not able to intraoperatively detect any emission of fluorescence of ICG, and in 3.4% (5/146) of the SLNs, fluorescence but not gamma emission was detected. There was a significant difference when comparing the detection tool used (gamma probe vs NIR optical laparoscopic camera) (*p* < 0.001). The topographic distribution based on the signal detected is shown in Fig. 5b.

In the *analysis of SLN detection by patient*, the radioactive component allowed the detection of SLNs in 97.1% (34/35) of the patients while the fluorescent component detected 80% (28/35). Among the 35 patients, in 20% (7/35) it was only one of the components and not both that allowed SLN detection. In 17.2% (6/35) of these patients, SLNs were detected only by the radioactive signal and in the remaining 2.8% (1/35) only one SLN with fluorescence emission was detected.

On comparing the SLNs pre-surgically visualized with those which were biopsied during surgery, there were no differences between the detection with SPECT/CT and surgical detection (*p* < 0.83), but there was a difference between planar and surgical detection and between planar and tomographic detection (*p* < 0.05).

In six women, more SLNs were biopsied during surgery than those visualized in the preoperative images, representing an increase in the number of lymphatic territories studied. Three patients with unilateral preoperative drainage presented bilateral SLNs; in one of these patients one paraaortic SLN was also biopsied. In two patients without drainage in the SPECT/CT, at least one unilateral SLN was biopsied, and in one of these patients paraaortic SLNs were also biopsied. In the remaining patient with unilateral pelvic drainage in the SPECT/CT, paraaortic SLNs underwent biopsy also.

### Histological lymph node results

Among the 35 patients in whom at least one SLN was biopsied, the histopathological study showed lymph node metastasis in 14.3% (5/35). Of these five affected patients, only one patient had metastatic paraaortic SLN. In this patient, pelvic metastatic nodes have also been detected.

In the other 30 patients with negative SLNs, no metastatic node was found in the lymphadenectomy sample. SLN detection by the TUMIR approach for detecting nodal involvement in patients in whom intraoperative SLNB was achieved were 100% (95% CI; 56.6–100%) for the sensitivity and PPV and 100% (95% CI; 88.6–100%) for the specificity and NPV.

In three of the 14 patients without preoperative drainage and in whom intraoperative detection was not achieved, metastatic lymph nodes were observed in the lymphadenectomy; in two patients the lymph nodes were of pelvic localization while in the other patient they were only paraaortic.

## Discussion

To date the value of hybrid tracer has not been evaluated in EC. In our series, this hybrid tracer was injected into the myometrium by the TUMIR approach for SLNB in patients with preoperative characteristics high- and intermediate-risk with the aim of improving lymphatic staging in this group of tumors.

The overall preoperative detection rate (69%) was less than that reported with radiotracer injected by the TUMIR approach (71–82%) [6, 20], as was the paraaortic preoperative detection rate of 36% compared to 41–45% described in previous studies, which were not, in any case, exclusively paraaortic. However, we found a higher rate of preoperative bilateral pelvic detection (56%) compared to the previously mentioned studies (29–37%).

The overall intraoperative detection rate was 71%, similar to that reported with radiotracer only injected by the TUMIR approach (74%) [20]. The intraoperative detection rate in the bilateral pelvic region was 57%, being superior than in previous TUMIR studies (29%), and in the paraaortic region SLNs were biopsied in 34%, similar to other studies published describing the TUMIR approach [20]. This increase in the detection rate of intraoperative bilateral pelvic SLN detection could be explained by the hybrid detection provided by this type of tracer since resection of the SLN is facilitated thanks to the visualization of elevated contrast enhancement provided by ICG after having performed the dissection of the fatty lymph tissue guided by the acoustic signal of the gamma detector probe by the radioactive signal.

There was an absence of preoperative drainage in 16 patients. In six women, there was a higher uptake in bone marrow or leakage to the peritoneum. The latter can be explained by the learning curve of different ultrasonographers, since this is an operator-dependent technique, and there is an association between ultrasonographer experience and the rate of preoperative SLN detection [6]. Among the 10 remaining patients showing an absence of drainage, lymph node infiltration was detected in the lymphadenectomy in three, and two of these three patients had pelvic nodes metastasis, which was interpreted as a metastatic blockage, while in the remaining patient lymph node metastasis was found in the paraaortic territory. According to the Memorial Sloan Kettering Cancer Center algorithm [21], this should not be considered as a false negative, since in the absence of drainage lymphadenectomy is recommended. However, in this patient the pelvic lymphadenectomy was negative, which would have led to non-completion of the paraaortic lymphadenectomy. This case highlights the importance of the study directed at the paraaortic lymphatic territory.

In two of the 16 patients without preoperative drainage, four SLNs were intraoperatively biopsied by radioactivity and fluorescence. SLNs with low activity may be undetectable on SPECT/CT but not during the intraoperative scan with gamma probe, which has proved to be more sensitive. Nonetheless, the SPECT/CT in our series increased the detection of SLN with respect to planar lymphoscintigraphy and correlates with surgical detection, as previously reported [22, 23].

In this study, the absence of preoperative drainage was almost 27%. The hybrid tracer has been used in cervical tumors with 100% preoperative drainage rates and pelvic bilaterality [19]. Therefore, it could be inferred that in contrast with other tumors, other factors could interfere with drainage and the absence of drainage in EC would not be attributable to the hybrid tracer. It is probably due to a multifactorial origin related to EC and this type of myometrial injection, in which the injection dose is inside a very compact and highly vascularized interstitial tissue [6].

The 68.5% of the SLNs biopsied were detected by both components. The remaining of the SLNs were identified only by the detection of one of the two components. The lack of detection of fluorescence (28.1% of the SLNs) could be explained by the limit of sensitivity of the NIR optical laparoscopic camera in vivo*,* lower than ex vivo detection, probably related to attenuation from the fatty tissue [17, 24]. In our series, we did not have a NIR camera for external detection to assess fluorescence [25]. The lack of radioactive detection (3.4%) might have been due to decay of the [^99m^Tc] obtaining a number of non-significant counts in vivo. In four of the five SLNs detected only with the fluorescence signal, the light emission was of great value since in one patient the drainage became bilateral, while in another patient without drainage at least one SLN was biopsied, and in another patient paraaortic SLNs were obtained. This demonstrates that the development of intraoperative fluorescence detector devices and the development and clinical implementation of new tracers are essential to improve and advance surgical efficacy. In addition, it cannot be ruled out that with TUMIR injection there may be a certain dissociation of the hybrid tracer which has not, to date, been described in other tumors and which may be related to the contact with blood, physiological saline and dilution in general. As a possible explanation, we hypothesize that it could be due to the injection being made in the myometrium, which is a highly vascularized structure probably with lesser volume of interstitial tissue and requires a greater volume of injected tracer. Also, some studies have analyzed the causes of the lower detection rate in this group of patients [26, 27]. For the TUMIR approach in particular, the association with older age, tumor size bigger than 2 cm and low radiotracer volumes have been demonstrated [6].We did not detect an association between BMI and a failure of detection as in other reported series [26].

Many studies have used ICG exclusively for visual identification of the SLN in EC, with detection rates greater than 96% [28, 29] allowing a high visual contrast between the SLN and the adjacent tissues, once the drainage basin has been identified. One of the drawbacks of ICG is that the fluorescence signal is easily attenuated by the surrounding fatty tissue, unlike the radiotracer signal. That is why, the radiotracer linked to ICG allows a quick and effective identification of the SLN during the search through the fatty tissue. Another of the limitations of the ICG is that it has a rapid diffusion through the lymphatic system and stains all the lymphatics structures of a territory, making it difficult to discern the true SLN since it is not always the first visible lymph nodes, which results in 7–8% of empty packets [30]. In addition, it impedes obtaining a preoperative lymphatic mapping.

In our series, the radioactive signal was the only detection tool that allowed the identification of SLN in more than 17% of patients, so we recommend not ruling out this component in ES surgeries.

For these reasons, we consider that the radioactive component of the hybrid tracer ([^99m^Tc]Tc-albumin nanocolloid) cannot be obviated since it provides the route map to achieve the target basin.

Once the drainage basin is identified, the high visual contrast provided by the ICG signal offers rapid intraoperative removal of the SLN. Therefore, it serves as a multimodal surgical guide to the true SLN in a single component, avoiding empty lymph node packets and even showing the less usual drainage territories [31]. Together, both components of this hybrid tracer are of special relevance in cases of uterine tumors due to the presence of a complex network of pelvic lymphatic vessels in which knowledge of drainage prior to surgery helps in surgical planning.

In the accuracy analysis of this study, the sensitivity and the NPV obtained for the detection of node metastasis were 100% in women with intermediate- and high-risk EC. These values are greater than those reported in previous studies in high-risk EC after cervical injection, with sensitivities ranging from 57 to 96.3% and a NPV of 83.3–99% [32–34] and are especially greater than hysteroscopic (58–100% and 89–100%, respectively) and myometrial injection (75–100% and 96–100%, respectively) [35]. The rate of SLN infiltration observed in our series was 14.3%. The current recommendations indicate that performing systematic pelvic and paraaortic lymphadenectomy implies overtreatment in up to 80% of patients who do not have lymphatic involvement. In addition, one recent study suggested that adding lymphadenectomy to the study of the SLN does not reduce the risk of relapse [36]. On the other hand, patients with paraaortic metastasis have worse oncological results than those with exclusive pelvic lymphatic involvement and, therefore, it is essential to evaluate the status of paraaortic SLN [37].

The present study has some limitations. Among these, this study was performed in a single center and therefore may lack external validity. Among other limitations, it was of note that it was not possible to perform ex vivo detection of fluorescence of the biopsied SLNs with a NIR optical camera optimized for the detection of fluorescence in room light, and therefore, we do not know if the in vivo nonfluorescent radioactive SLNS would have been fluorescent by ex vivo stimulation with this another camera. The reduced number of patients is another limitation. However, one of the main strengths of this study was that it was a homogeneous group of patients with intermediate- and high-risk EC and all the surgeries were performed by the same surgical team. In addition, in the present study, all the patients underwent complete pelvic and paraaortic lymphadenectomy up to the renal vessels, in contrast with other studies evaluating the validity of SLNB in high-risk EC [38, 39].

Although the percentage of patients with drainage who could undergo intraoperative detection of the SLN was low (around 70%), the low rate of false negatives is promising. However, multicenter studies are needed to confirm these results and validate that this procedure reduces morbidity, surgical time and costs. As the hybrid tracer injected via TUMIR provides information on the status of paraaortic lymph nodes, based on the recommendations of the clinical guidelines, the strategy to increase the number of patients undergoing intraoperative SLNB would be to inject the hybrid tracer into the cervix in patients without drainage. This would at least provide a map of pelvic lymphatic drainage and thereby avoid lymphadenectomy.

To our knowledge, this is the first study on the use of the hybrid tracer for the detection of SLNs in EC, which, furthermore, is injected via TUMIR. Our results of the use of this hybrid tracer confirm the feasibility of this procedure and are consistent with those described in prostate and cervical cancer [17, 19]. The hybrid tracer adds the benefits of presurgical planning with a dual intraoperative guidance, allowing a potential increase in SLN detection rate (up to 20% of patients in our series).

## Conclusion

SLNB with a hybrid tracer injected by the TUMIR approach is feasible in intermediate- and high-risk EC. The hybrid tracer allows, in a single injection, the well-established benefits of the standard radioguided procedure and the visualization of SLNs by fluorescence imaging. In patients with depicted lymphatic drainage on preoperative SLN imaging, the use of hybrid tracer by TUMIR injection achieves an elevated paraaortic detection rate and allows a potential increase in SLN detection. Notwithstanding, based on our findings, the radioactive component of the hybrid tracer ([^99m^Tc]Tc-albumin nanocolloid) cannot be obviated.

## Data Availability

The datasets generated during and/or analyzed during the current study are available from the corresponding author on reasonable request.
